# Solute‐Induced Modulation of Hydrogen Bonding, Ion Hydration, and Protonation in Hydrogels for Enhanced Microwave Absorption

**DOI:** 10.1002/advs.78744

**Published:** 2026-11-09

**Authors:** Zijing Li, Xiangdong Wang, Shengchong Hui, Gaoxu Zhang, Jiaqi Wang, Wei Zhou, Hongjing Wu, Limin Zhang

**Affiliations:** ^1^ MOE Key Laboratory of Material Physics and Chemistry under Extraordinary Conditions School of Physical Science and Technology Northwestern Polytechnical University Xi'an People's Republic of China; ^2^ Hunan Key Laboratory of Applied Environmental Photocatalysis Changsha University Changsha People's Republic of China; ^3^ State Key Laboratory of Powder Metallurgy Central South University Changsha People's Republic of China; ^4^ Sanhang Science & Technology Buliding Shenzhen Research Institute of Northwestern Polytechnical University Shenzhen Guangdong People's Republic of China

**Keywords:** dielectric loss, hydrogel, microwave absorption, noncovalent interaction

## Abstract

Hydrogels have emerged as promising candidates for microwave absorption in next‐generation flexible electronics and soft robotics, whose rapid development demands increasingly sophisticated and tunable dielectric properties from microwave‐absorbing materials. However, the coupling of hydrogen bonding, ionic coordination, and protonation involving water creates a complex noncovalent interaction network, making it difficult to explore the main contribution of each interaction to the dielectric response, which hinders modulation of dielectric performance. Here, we propose a solute‐mediated regulation strategy based on acrylic acid (AA) and acrylamide (AM) backbones by introducing polar groups, metal salts (M^+^), and acidic solutions (H^+^) to systematically investigate hydrogen bonding, metal‐ion hydration, and protonation in regulating dielectric properties. The results reveal that hydrogen bonding and ionic coordination suppress proton hopping while generating new dipolar polarization units, thereby reducing reflection and shifting the dielectric response from conduction‐dominated to polarization‐dominated, whereas protonation balances conduction and polarization by regulating water conduction and suppressing hydrogen‐bond‐induced orientational polarization. Notably, hydrogen bonding coupled with Co(NO_3_)_2_‐induced hydrated ions and tartaric acid‐induced protonation both enabled a broad effective absorption bandwidth (EAB) of 7.1 GHz at thicknesses of 1.88 and 1.85 mm, respectively.

## Graphical abstract

Systematic solute screening reveals how noncovalent interactions regulate dielectric loss in P(AA‐co‐AM) hydrogels. By tuning proton conduction, ion hydration, and hydrogen‐bond interactions, the dielectric response can be precisely controlled, enabling tailored hydrogels with broadband microwave absorption and providing a general strategy for designing flexible electromagnetic absorbers.

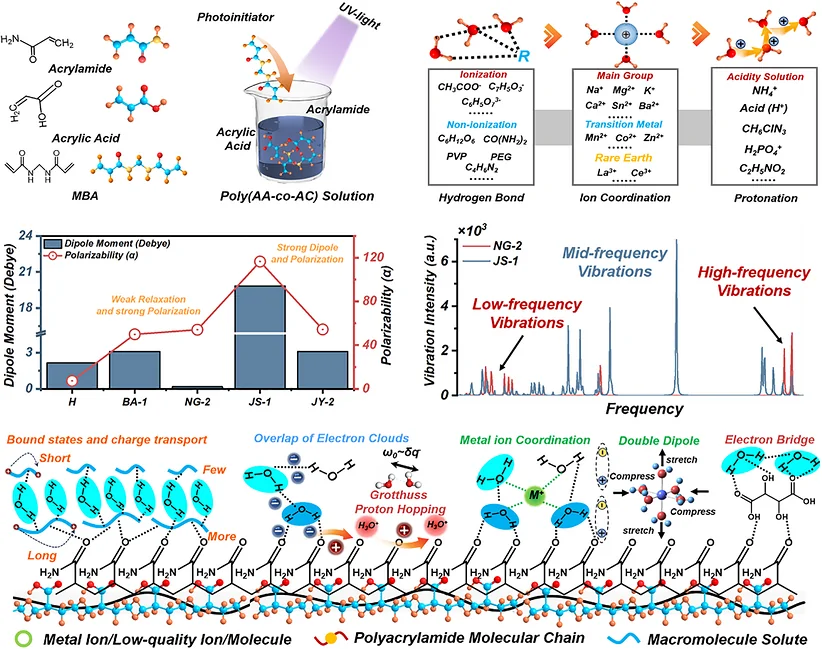

## 1 Introduction

The rapid development of artificial intelligence (AI) and soft robotics is driving the evolution of information systems, while presenting new challenges for electromagnetic security and placing increasingly stringent demands on flexible electromagnetic wave absorbing materials [1, 2, 3, 4, 5, 6, 7, 8]. The dielectric response of hydrogels is closely related to charge behavior within their highly hydrated networks [9, 10, 11, 12], where hydrogen bonding, ion hydration, and protonation of water molecules can regulate dielectric properties by altering local molecular structures, charge transport, and dipole reorientation [13, 14, 15, 16]. However, these noncovalent interactions typically coexist and are strongly coupled within hydrogels, making it difficult to fully resolve the individual contribution of each interaction to the dielectric response and its regulatory capacity, thereby hindering further breakthroughs in dielectric performance. Therefore, there is an urgent need for new paradigms of dielectric regulation that exploit noncovalent interactions between water molecules and metal ions, polar groups, and protons to construct specific localized charge configurations and thereby regulate the electrical conductivity and polarization characteristics of hydrogels.

To overcome this bottleneck, three types of noncovalent interactions need to be established within the hydrogel network: hydrogen bonds constructed by highly electronegative atoms, hydrated ions constructed by cationic coordination, and protonated water constructed by an acidic environment, thereby providing a new theoretical framework for modulating the dielectric behavior of hydrogels. (1) By exploiting the confinement effect of polymers on water, highly electronegative groups are introduced into the aqueous phase space, and the differences in electrostatic interactions between different groups and water are used to construct hydrogen bonds of varying strength, achieving rearrangement of water molecules and inducing polarization deformation of electron clouds, thereby creating new ion channels and polarization sites. (2) By utilizing the lone‐pair electrons of oxygen atoms, metal cations with vacant valence orbitals are introduced to form O─M bonds, and the differences in their electronic orbitals and hybridization modes are exploited to construct hydrated ions with different hydration forms and strengths, producing localized confinement of water, altering charge transport between water molecules, and constructing entirely new polarization units centered on metal ions. (3) By exploiting the intrinsic charge transport mechanism of water, the degree of protonation of water molecules and hydrogen‐containing groups is altered through H^+^ or an acidic environment, achieving enhancement of intrinsic proton hopping of water molecules and optimization of ion channels. The charge rearrangement and center shifts induced by these noncovalent interactions construct new conduction pathways and dipole polarization, thereby achieving modulation of the dielectric behavior within hydrogels.

To establish this theoretical framework, we employ an AA‐co‐AM polymer backbone with high solution compatibility and directly construct three types of noncovalent interactions involving water molecules by introducing different soluble solutes [17, 18, 19]. Because these solutes differ substantially in functional group polarity, ionic species, and acidity or basicity, they can greatly expand the electronic configurations of the hydrogel and generate noncovalent interactions with distinct strengths and forms [20, 21, 22, 23, 24, 25, 26, 27]. First, hydrogen bonding originates from the electrostatic attraction between electronegative atoms and hydrogen atoms. Accordingly, polar groups with different chemical identities and concentrations (OH, NH_2_, COOH, C═O, etc.) can be introduced to establish hydrogen bonds with tunable strengths. This approach enables us to elucidate how charge redistribution between functional groups and water regulates ionic conductivity and dipolar polarization in hydrogels. Second, the local coordination of hydrated metal ions is governed by interactions between the valence orbitals of metal ions and the lone pair electrons of oxygen. The distinct energy levels of the valence s, p, d, and f orbitals across main group, transition metal, and rare earth ions (Mg^2+^, Co^2+^, Zn^2+^, Ba^2+^, La^3+^, etc.) allow hydrated ions with markedly different coordination strengths and local structures to be constructed. These differences can directly regulate charge transport between water molecules and the polarization response of hydrated ions. Third, the protonation of water molecules and hydrogen‐containing functional groups is fundamentally determined by their degree of ionization in an aqueous environment. By varying the H^+^ concentration using different acidic solutes (HCl, HNO_3_, H_4_PO_4_, C_4_H_6_O_6_, etc.), the hydrogen bonding network and proton hopping process can be systematically modulated [28, 29, 30, 31, 32, 33, 34, 35, 36].

Here, based on the aforementioned theoretical and experimental framework, 127 systems of water noncovalent interactions were successfully constructed with acrylamide and acrylic acid matrices. By constructing a series of hydrogels from polar functional groups, metal ions, acidic solutions, and AA/AM polymer backbones, controllable hydrogen bonding, metal ion hydration, and protonation involving water can be introduced into the polymer network, thereby inducing charge redistribution. When organic molecules with multiple polar groups are introduced, as hydrogen bonding interactions increase, their orienting effect gradually masks their polarization deformation of electron clouds, causing water molecules to rearrange and become confined, thereby constructing new charge transport modes and restricting the polarization of water. For example, from the single‐slope Mott–Schottky (M‐S) curve of the pure hydrogel to the double‐slope M‐S curve of the polyethylene glycol gel, with the reduction of the permittivity polarization (−1.99). When metal ions are introduced, as the valence electron orbitals rise, the polyhedral structures induced by coordination between their empty orbitals and oxygen atoms suppress the intrinsic charge transport of water molecules and form localized polarization units. In particular, benefiting from the Jahn–Teller effect of the Co^2+^ *d*‐orbitals, the distortion of the hydrated ion structure leads to large‐scale charge displacement and forms new strong polarization units, manifested as strong suppression of the permittivity conduction (−2.25) and enhancement of permittivity polarization (+1.02). When acidic solutions are introduced, with the action of H^+^, the degree of protonation of water molecules and groups increases, and the charge transport efficiency rises. Notably, the hydrogel with tartaric acid, through the highly ionized H^+^ and the polar groups of C_4_H_4_O_6_^2−^, simultaneously achieves the strengthening of proton hopping conduction and the construction of local dipoles, manifested as a double‐slope M‐S curve and simultaneous enhancement of permittivity polarization (+0.95) and conduction (+0.61). Therefore, coupling *d*‐orbital‐induced hydrated ions (Co(NO_3_)_2_) with backbone hydrogen bonding and acid‐induced (tartaric acid) water protonation with free polar‐group hydrogen bonding afforded an ultrawide EAB of 7.1 GHz at thicknesses of 1.88 and 1.85 mm, respectively. This study elucidates the correspondence between hydrogen bonding, hydrated ions, protonation, and dielectric behavior, providing a complete feasible pathway and theoretical support for improving the dielectric performance of hydrogel systems.

## 2 Results and Discussion

### 2.1 The Design and Preparation of Hydrogels With Various Solutes

To gain deeper insight into the dielectric response of hydrogels with different solvents, it is essential to first design a gel network with broad solute compatibility and high architectural flexibility, thereby ensuring effective polymerization and tunable electromagnetic response. Therefore, we chose soft acrylamide (AM) as the backbone monomer for its favorable compatibility mediated by intermolecular hydrogen bonding, and introduced acrylic acid (AA) as a comonomer [37, 38, 39], whose pendant carboxyl groups can adaptively undergo protonation or ionization in response to pH variations caused by different salt solutions, thereby preserving the structural integrity of the gel network while ensuring a localized charge response. Bifunctional N,N′‐methylenebisacrylamide (MBA) was used as a crosslinker to suppress linear chain propagation and construct a robust 3D copolymer network (AM‐co‐AA) (Figure 1a). Furthermore, contour plane maps of the highest occupied molecular orbital (HOMO) and lowest unoccupied molecular orbital (LUMO) reveal enhanced electron wavefunction localization at the pendant groups of the network, providing excellent sites for charge migration upon subsequent solute introduction and further enriching the dielectric response ability of the system (Figure 1b).

**FIGURE 1:**
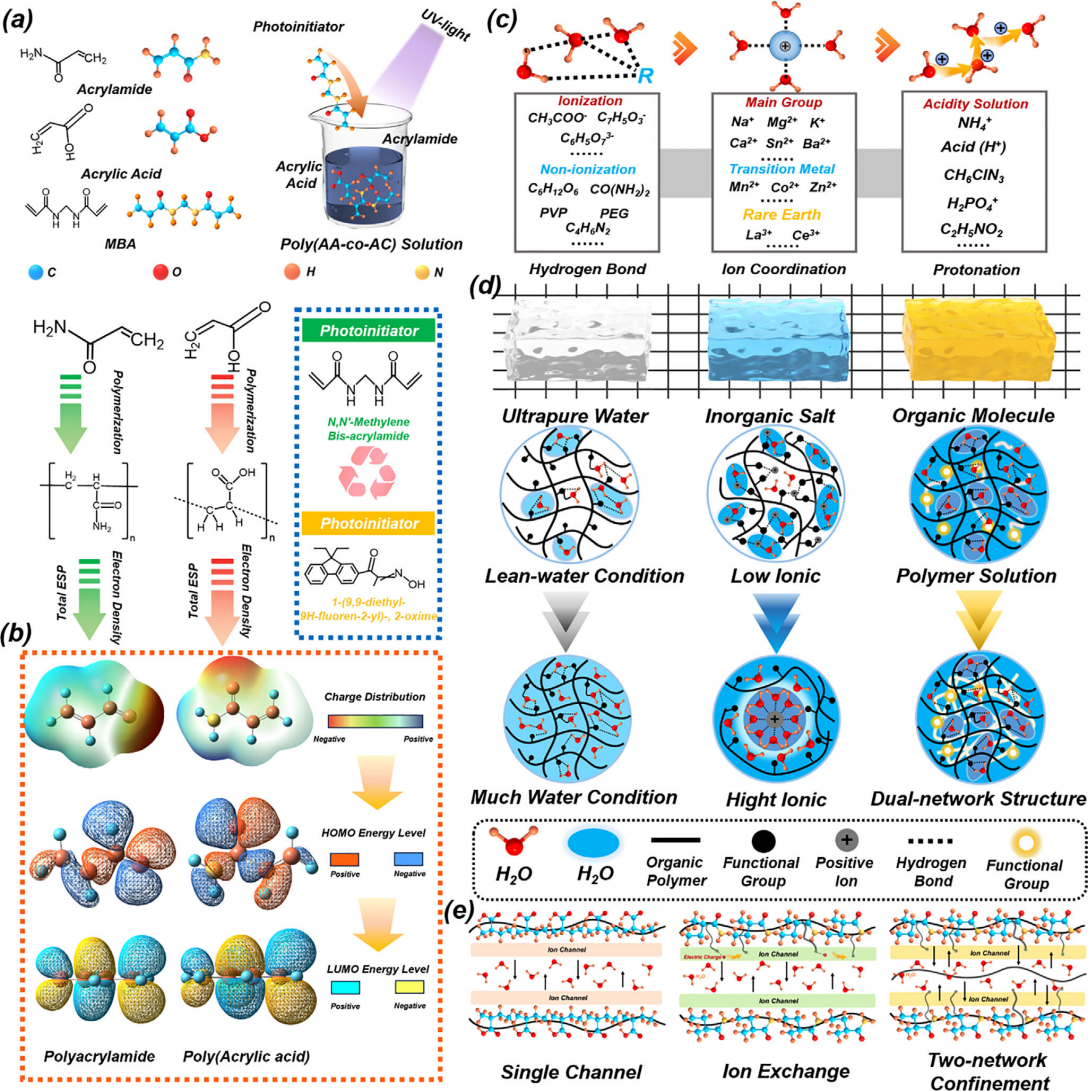
(a) The polymerization pathway of hydrogels. (b) The charge distribution, HOMO energy level, and LUMO energy level of Polyacrylamide and Acrylic Acid. (c) The classification of noncovalent interactions formed by different solutes. (d) The disparate states of water and ions in the gel network. (e) The different charge transport paths in different solution states.

Based on the P(AM‐co‐AA), we designed a series of inorganic salt gels and organic molecule gels based on three types of noncovalent interactions, namely hydrogen bonding, ionic hydration, and protonation of water, by introducing solutes with distinct characteristics to modulate the water coordination state, ionic conductivity, and charge distribution on the pendant groups of the polymer chains. (Figure 1c and Table S1). For inorganic salts, the coordination of metal ions is governed by the competition between carboxylate groups from Lewis bases and free water. Main‐group metal ions with small ionic radii primarily neutralize ─COO^−^ through electrostatic interactions, tending to form a loose hydration layer that minimally perturbs the network structure. In contrast, transition metal ions with large ionic radii and high valence states form ionic crosslinking sites with pendant ─OH and ─COOH groups. As their concentration increases, these transition metal ions extensively coordinate with water molecules to adopt polyhedron coordination structures. Owing to the distinct spatial orientation of *d*‐orbitals among different transition metals, the resulting crystal field stabilization energies differ, giving rise to diverse stacking modes of water‐coordination bonds [40, 41, 42]. For water‐soluble organic molecules, at low concentrations they are dispersed and tend to coordinate with side functional groups of the polymer backbone, thereby restricting charge transport or creating polarization sites. At high concentrations, they preferentially form self‐assembled micelles driven by intermolecular interactions, which further partition the conductive regions of free water molecules (Figure 1d,e).

In summary, the polymer network is dielectrically inert and mainly serves as a 3D framework that confines water and accommodates diverse solutes. Solutes modulate the coordination structure and distribution of water molecules as well as ion transport pathways, with distinct water configurations contributing to dipolar polarization and ion hopping along polymer chains governing conduction loss. Consequently, variations in solute type and concentration regulate the balance between polarization and conduction, enabling tunable electromagnetic responses across different frequency bands. A broad range of samples is therefore essential for correlating electromagnetic loss mechanisms with specific water coordination environments and distributions.

### 2.2 Characterization of Chemical Bonds and Microstructures

To clarify how noncovalent interactions and charge distributions regulate the dielectric properties of the gels, we characterized the coordination states of N and O by XPS (Figures S1–S5). The pristine hydrogel (H) shows a dominant N 1s peak at ∼399.5 eV from π‐conjugated amine groups and a minor protonated amine peak at ∼401.5 eV, while the O 1s spectrum contains nearly equal contributions from single‐bonded O (∼533.0 eV) and double‐bonded O (∼531.5 eV). Upon introducing Co^2+^, coordination with amide C═O groups reduces the electron density of O and, through the polymer backbone, shifts the amine and C═O peaks to higher binding energies: the N 1s peak shifts by 0.12 and 0.04 eV for NG‐2 and BA‐1, respectively, while the double‐bonded O peak shifts by 0.27 and 0.10 eV. These shifts indicate Co^2+^─O coordination, enhanced amine protonation, and immobilization of free water, favoring suppressed ionic conduction and localized charge polarization. In contrast, PEG (JY‐2) coordinates only through terminal ─OH groups and ether linkages without appreciable XPS shifts, whereas tartaric acid (JS‐1) introduces H^+^ and COO^−^, increasing amine protonation and shifting the main N 1s and O 1s peaks to higher binding energies (Figure 2a,b). Mott–Schottky analysis further shows that Co^2+^ reverses the charge‐transport type, attributed to positive‐charge pinning by strong Co^2+^─O coordination, while the opposite trend in organic‐molecule systems confirms that different ion coordination modes govern distinct charge‐transport mechanisms (Figure 2c and Figures S6 and S7) [43, 44].

**FIGURE 2:**
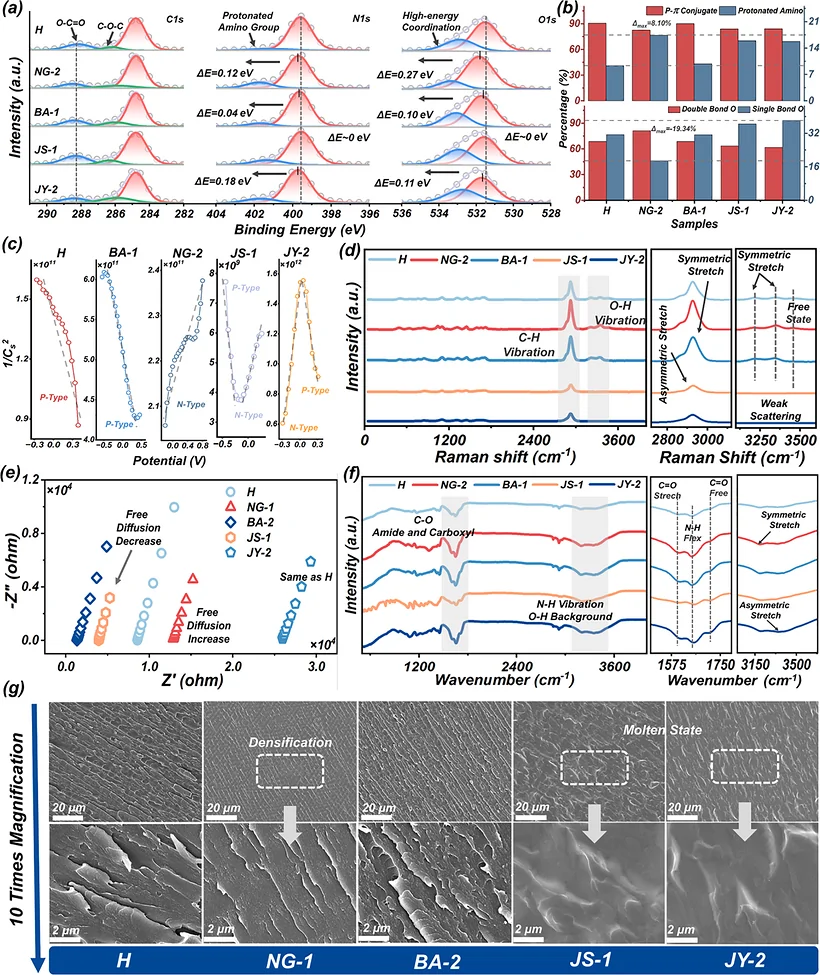
(a) The XPS fine spectra of the C1s, N1s, and O1s of the hydrogel. (b) The chemical bond percentages of P‐π conjugate, protonated amino, O─O and O═O of hydrogels. (c) The Mott–Schottky curves and carrier types of hydrogels. (d) The Raman spectroscopy and local magnification of samples. (e) The electrochemical impedance spectroscopy (EIS) of hydrogels. (f) The infrared absorption spectrum and local magnification of samples. (g) The scanning electron microscope (SEM) images at 20 and 2 µm of hydrogels.

To corroborate the effects of chemical bonding and the polymer network on the dielectric response, we characterized the gels by Raman, FTIR, and electrochemical impedance spectroscopy (EIS) (Figure 2d–f and Figures S8 and S9). Raman spectra show C─H stretching at 2800–3000 cm^−1^ and bound O─H stretching at 3200–3500 cm^−1^, while FTIR spectra exhibit C═O vibrations at 1600–1700 cm^−1^ and N─H vibrations at 3100–3500 cm^−1^. Transition‐metal‐ion introduction enhances C─H, C═O, N─H, and O─H vibrations, indicating strengthened molecular orientation and metal‐ion hydration with polymer groups and water, whereas H^+^ mainly enhances N─H vibrations. In contrast, non‐ionizable soluble macromolecules induce negligible changes in the polymer electronic structure but weaken asymmetric O─H and N─H vibrations by segmenting free water. The EIS results further confirmed that ions effectively regulate the charge‐transfer resistance and thereby modulate the electrical properties of the system, whereas the molecular chains have only a minor influence on it. SEM images (20 and 2 µm, Figure 2g) show dense layered structures for H and BA‐2, whereas Co^2+^ coordination promotes stronger water clustering and produces a denser NG‐1 network by reducing free and weakly bound water. Conversely, PEG (JY‐2) and tartaric acid (JS‐1) induce localized water segregation and altered coordination environments, resulting in more porous 3D networks [45].

In summary, compared with pristine H, NG‐2 and JS‐1 regulate dielectric responses through distinct solute‐mediated mechanisms. In NG‐2, strong Co^2+^ coordination with C═O and ─OH groups induces electron‐cloud rearrangement and localized dipole polarization, while immobilizing free water and suppressing long‐range ion migration. Coordination‐induced positive‐charge pinning may further promote hole‐like transport and modify space‐charge polarization. In contrast, JS‐1 introduces H^+^ and COO^−^, promoting amine protonation and dipole polarization while modulating charge transport to balance conductive and polarization losses. These effects differ from H, whose dielectric response is primarily governed by proton transport through the water‐mediated hydrogen‐bond network and the associated dipolar relaxation. Meanwhile, non‐ionizable organic molecules regulate dielectric behavior mainly by segmenting the free‐water phase, weakening asymmetric O─H vibrations, and disrupting ion‐transport pathways. Collectively, these distinct mechanisms provide a basis for rationally tuning hydrogel dielectric properties through solute regulation.

### 2.3 Permittivity Modulation via Polarization and Conduction Behaviors

Based on the above results, we analyzed the dielectric properties of gels using complex permittivity (ε′, ε″) and permeability (µ′, µ″). Because the gels contain no effective magnetic components, µ′≈1 and µ″≈0, and magnetic loss is negligible (Figure S10). At a fixed free‐charge concentration of 0.5 mol/L, varying solute molecular mass by up to 2412% caused no linear change in dielectric properties, indicating that molecular mass is not the dominant factor, whereas cation species exerted a pronounced influence (Figures S11–S33). In particular, the modulation by transition metal cations arises from the difference in spin states between high‐energy and low‐energy *d*‐orbital electrons, and their coordination with highly electronegative atoms follows specific stacking patterns, leading to a highly bound state for water molecules and pendant groups. This is further reflected in the observation that, at the same charge concentration, the main‐group metal salt (NaCl) and the pure hydrogel exhibited nearly identical polarization loss and conduction loss, whereas the transition metal salts (Co(NO_3_)_2_) showed localized suppression of ionic conduction and a substantial increase in polarization loss (Figure 3a–c). In brief, they demonstrate enhanced polarization loss and localized regulation of charge transport, making them particularly suitable for impedance modulation in the X and Ku bands.

**FIGURE 3:**
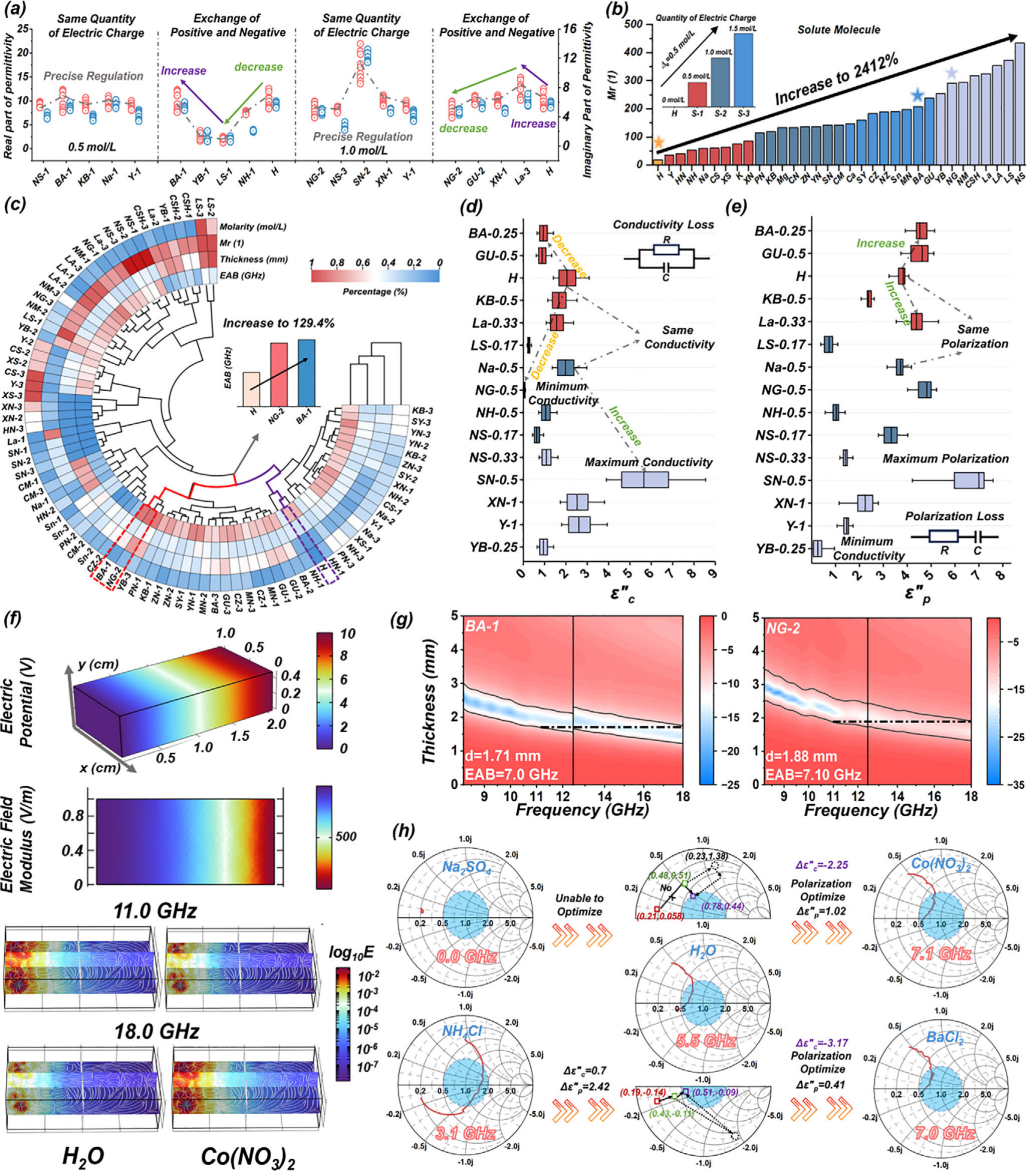
(a) The comparison of the complex permittivity of anionic and cationic gels at identical charge densities in an inorganic salt system. (b) The relative molecular mass of the inorganic salt system. (c) The circular heat map of molarity, relative molecular mass, EAB, and thickness (*d*) in the inorganic salt system. (d‐e) The average values of ε_c_″ and ε_p_″ for the inorganic salt system. (f) The electric potential and electric field modulus of H and NG‐2 at 11.0 and 18.0 GHz. (g) The 2D mapping of reflection loss for BA‐1 and NG‐2. (h) The Smith chart for impedance matching controlled by capacitance/inductance.

Furthermore, the decomposition of the imaginary part into conduction loss and polarization loss revealed distinct patterns: (1) Metal cations all suppressed conduction loss to varying degrees, with transition metal and rare earth cations exhibiting particularly strong suppression, whereas nonmetal cations promoted conduction loss. (2) Metal cations and heavy main‐group metal ions markedly enhanced polarization loss, while main‐group metal ions generally caused a decline. Notably, Ba(CH_3_COO)_2_ showed the lowest polarization loss, which may be attributed to CH_3_COO^−^ promoting the ionization of the PAA pendant ─COO^−^ groups, further neutralizing the cations and enhancing the contraction of the gel network. (3) In particular, NaCl exerted negligible influence on the conduction and polarization behavior of the gel at high frequencies, whereas NH_4_Cl exhibited an anomalous suppression of conduction loss. This may originate from the strong hydration ability of NH_4_^+^ and the high hydration enthalpy induced by the strong salting‐out capacity of SO_4_^2−^. (4) In addition, the influence of anions on the dielectric properties is primarily manifested as a coupling effect with cations. The key lies in the fact that the introduction of anions alters the environmental pH or introduces new functional groups, thereby changing the coordination environment and affecting the coordination of cations with the polymer network and free water molecules. Consequently, the effects of anions and cations on conduction and polarization can shift with variations in ion concentration, thus influencing the overall impedance matching. At low concentrations, both conduction and polarization are generally suppressed; as the concentration increases, the loss gradually intensifies, ultimately achieving excellent impedance matching (NG‐2 and BA‐1) with a bandwidth reaching 7.1 and 7.0 GHz, thicknesses of 1.71 and 1.88 mm (Figure 3d,e).

To elucidate how inorganic salts improve impedance matching, COMSOL simulations were used to map the potential difference and electric‐field intensity from 11.0 to 18.0 GHz (Figure 3f and Figure S34). The reversal in electric potential difference and field intensity between H and NG‐2 from 11.0 to 18.0 GHz indicates that Co^2+^ suppresses the intermediate‐frequency response of water while extending the electromagnetic response toward higher frequencies. Smith‐chart analysis further highlights the distinct dielectric‐regulation mechanisms of H, NG‐2, and BA‐1 (Figure 3g,h). At 18.0 GHz, 0.25 mol/L Na_2_SO_4_ altered the intrinsic conduction mode, whereas NH_4_Cl suppressed both conduction (−0.7) and polarization (−2.42), shifting the impedance outside the effective matching region. Compared with pristine hydrogel (H), NG‐2 exhibited suppressed conduction (−2.25) and enhanced polarization (+1.02) at 11.0 GHz due to Co^2^^+^ incorporation, whereas BA‐1 showed a stronger suppression of conduction (−3.17) and a more moderate enhancement of polarization (+0.41) at 18.0 GHz following Ba^2^^+^ incorporation. These distinct responses enable NG‐2 and BA‐1 to improve impedance matching in different frequency bands. Overall, the insufficient mid‐frequency polarization and pronounced high‐frequency skin effect limit the absorption performance of H, while Co^2+^ and Ba^2+^ regulate the balance between conduction and polarization losses through different ionic interactions, thereby facilitating impedance matching and broadening the effective absorption bandwidth.

The electromagnetic properties of soluble‐organic‐molecule gels were characterized similarly (Figures S35–S53). At the same solute concentration, acidic gels generally exhibited lower electromagnetic parameters than H, with a clear dependence on H^+^ concentration; notably, the HCl‐containing YS gel showed the lowest imaginary permittivity. This indicates that high H^+^ concentrations enhance amine protonation and alter hydrogen‐bond association, strengthening free‐water binding and suppressing hydrogen‐bond‐induced polarization. Although molecular mass was not the dominant determinant, organic solutes generally reduced the dielectric response, and increasing molecular mass further decreased the dielectric parameters in most systems. In particular, high molecular weight PVP (∼58 000; PVP‐2‐2) exhibited a much lower dielectric response than PVP (∼10 000) (Figure 4a–c). Increasing the concentration of low‐molecular‐weight PVP slightly increased permittivity, whereas high‐MW PVP reduced ε″ by strongly suppressing conduction, leaving polarization as the main loss contribution. These results demonstrate that long organic chains mainly segment and immobilize water, thereby suppressing conduction and reducing polarization, unlike metal ions, which can simultaneously introduce additional polarization and regulate conduction. Thus, organic solutes mainly modulate dielectric properties through the pre‐existing water framework without creating new charge‐transport or polarization modes (Figure 4d,e).

**FIGURE 4:**
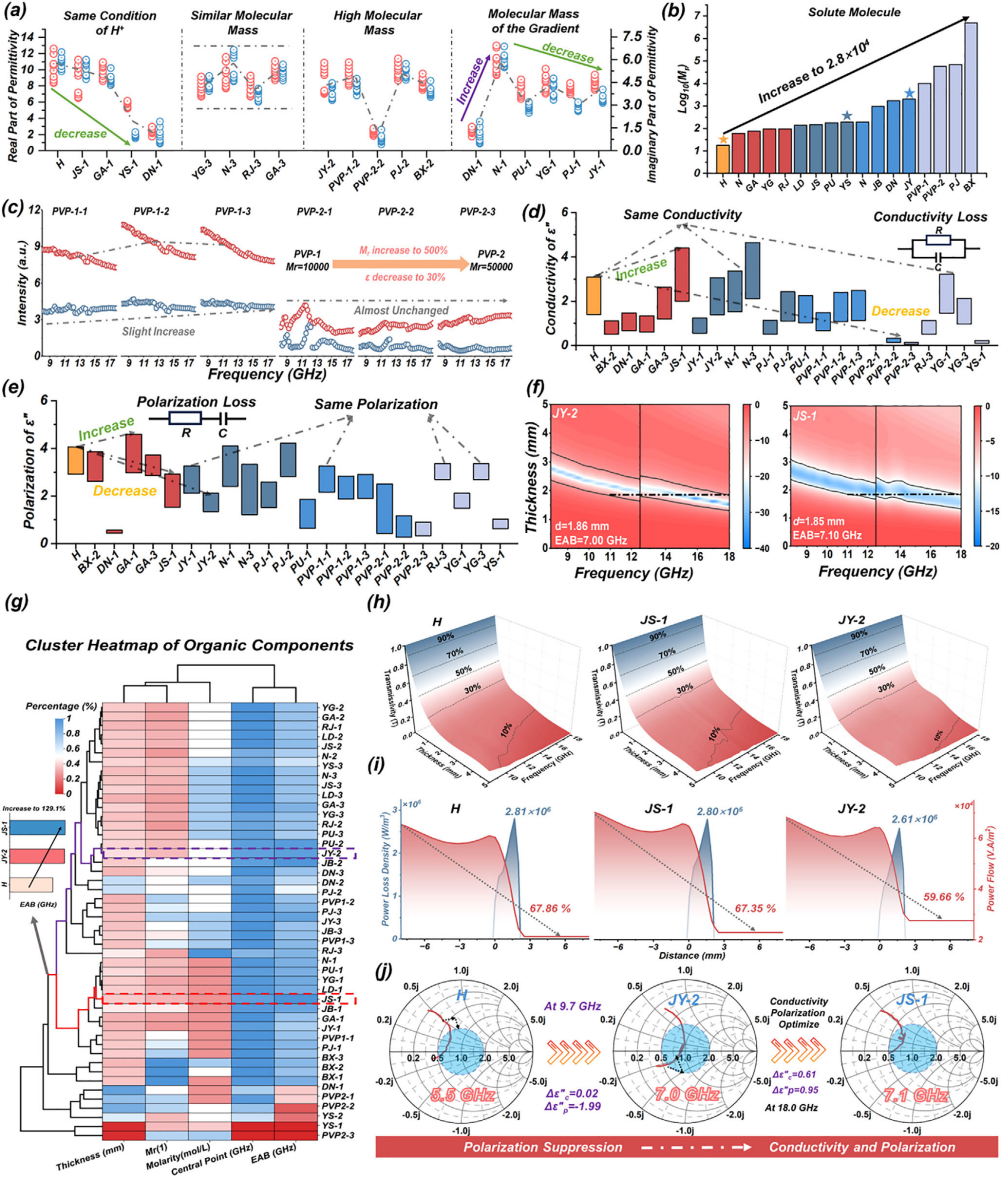
(a) The comparison of the complex permittivity of pH and molecular mass in an organic molecule system. (b) The relative molecular mass of the organic molecule system. (c) The complex permittivity of PVP hydrogels at identical mass loading for varying molecular weights. (d,e) The matching thickness, central frequency points, and average values of ε_c_″ and ε_p_″ for the organic molecule system. The 2D map of reflection loss for JY‐2 and JS‐1. (g) The cluster heatmap of molarity, relative molecular mass, central point, EAB, and thickness (*d*) with the organic molecule system. (h,i) The 3D transmittance map at 8.2–18.0 GHz, power loss density, and power flow of H, JS‐1, and JY‐2. (j) The Smith chart for impedance matching controlled by capacitance/inductance.

To further assess the microwave absorption of soluble‐organic‐molecule gels, we correlated molar concentration, molecular mass, center frequency, EAB, and thickness (Figure 4f,g). Most organic‐solute gels exhibited lower EAB than H (5.5 GHz, at a thickness of 1.85 mm), with weakly interactive solutes generally reducing the EAB to below 3.0 GHz. In contrast, tartaric acid (JS‐1), which provides low‐concentration H^+^ and abundant hydrogen‐bonding sites, and high‐molecular‐weight PEG (JY‐2), containing ether oxygen groups, achieved ultrawide EABs of 7.1 and 7.0 GHz at a thickness of 1.76 and 1.85 mm, respectively, reaching 129.1% of H [46, 47]. CST simulations without a metal backplane showed nearly identical transmission and power‐loss characteristics for H and JS‐1, while JY‐2 exhibited slightly higher transmittance and lower loss, indicating comparable direct attenuation for H/JS‐1 but weaker loss for JY‐2 (Figure 4h,i). Smith‐chart analysis further showed that near 9.7 GHz, JY‐2 approached the matching region by suppressing polarization loss by 1.99 while barely changing conduction loss (0.02); near 18.0 GHz, JS‐1 approached impedance matching through enhanced conduction (0.61) and polarization loss (0.95) (Figure 4j). These results indicate that the enhanced EAB of JS‐1 and JY‐2 mainly arises from improved impedance matching through distinct regulation of polarization and conduction.

### 2.4 Electromagnetic Loss Mechanism of Inorganic and Organic Systems

To further elucidate how noncovalent interactions induced by solute molecules govern the dielectric behavior of hydrogels, it is essential to clarify how hydrogen bonding, ion hydration, and protonation of water molecules influence the charge states and charge dynamics within the hydrogel network [48, 49, 50, 51, 52, 53]. The attenuation constants and electromagnetic field attenuation capabilities of the gels without a backing plate were directly calculated from the measured electromagnetic parameters. The results showed that the differences in direct attenuation among the gels were relatively small. Specifically, BA‐1 exhibited the highest direct attenuation rate of 76.5%, whereas JY‐2 showed the lowest value of 72.9%, corresponding to a maximum difference of only 3.6%. (Figure 5a,b). This indicates that the differences in high‐frequency transmittance of hydrogel systems under the metal‐backplane model are small, and the variation in EAB may primarily originate from reflection caused by the surface skin effect, internal loss within the gel, and the proportion of dielectric relaxation. Cole–Cole plots reveal that the Debye relaxation shifted toward higher frequencies upon the introduction of Co^2+^ and tartaric acid, with JS‐1 exhibiting the largest shift, which further corroborates that the influence of Co^2+^, H^+^, and ether oxygen on water molecule coordination leads to a transition in polarization (Figure 5c). Furthermore, we measured the carrier concentration of the gels using Mott–Schottky curves and found that the pure hydrogel possesses a far higher carrier concentration than the other gels, while the gel containing polyethylene glycol as the solute exhibits the lowest carrier concentration (Figure 5d). This further confirms that the immobilization of free water molecules by metal ions directly impacts the overall conductivity of the gel and weakens the transport efficiency of charge carriers, whereas the segmentation of free water molecules by polyethylene glycol disrupts the continuity of the original ion transport channels within the gel and creates new, small‐scale localized transport. Both states of water suppress the overall conduction loss and weaken the reflection caused by the skin effect on the gel surface, confirming the reduction in the conduction component of the imaginary part of the permittivity. Notably, the introduction of Co^2+^ and Ba^2+^ weakened the compression effect on electromagnetic waves inside the gel, with their propagation capability markedly enhanced, allowing more electromagnetic waves to enter the gel interior and reducing surface reflection. However, a strong compression of the phase velocity is still retained, remaining below 0.4c in all cases, indicating sustained strong dielectric relaxation capability (Figure 5e). In terms of microstructural changes, the high‐spin Co^2+^, benefiting from the high energy state arising from the occupation of high‐energy *d*‐orbitals, undergoes a Jahn–Teller distortion upon coordination with H_2_O. The distributions of HOMO, LUMO, and spin density further reveal that this distortion leads to an axial elongation of the octahedral coordination geometry of water molecules, causing a weak separation of local positive and negative charges and forming a unique localized dipole (Figure 5f). Owing to the weak interaction, this localized dipole possesses a polarizability far higher than that of water molecules, resulting in vibrational modes in both the low‐frequency and high‐frequency regions. In contrast, the strong noncovalent hydrogen‐bonding interactions between tartaric acid and water molecules generate a larger dipole moment and the highest polarizability in the mid‐frequency band, along with strong vibrational modes distinct from those of Co^2+^. These structural changes, together with the gel conductivity, indicate that the localized water coordination induced by metal ions and the strong hydrogen‐bonding structures introduced by pendant groups greatly enhance the dielectric polarization behavior of the gel and shift the frequency range of dipolar polarization (Figure 5g,h). Moreover, from the influence of macromolecules on the charge distribution of the gel, it is evident that ionizable organic molecular structures exhibit a stronger surface charge density, while the surface charge density of non‐ionizable macromolecules is nearly identical to that of water molecules. This implies that ionizable or strongly polar organic molecules are expected to provide more efficient channels for charge transport. Given that the conduction of water molecules relies on proton hopping along the backbone, highly polar short molecular chains can further reduce the barrier for localized proton hopping, thereby strengthening the conduction loss (Figure 5i).

**FIGURE 5:**
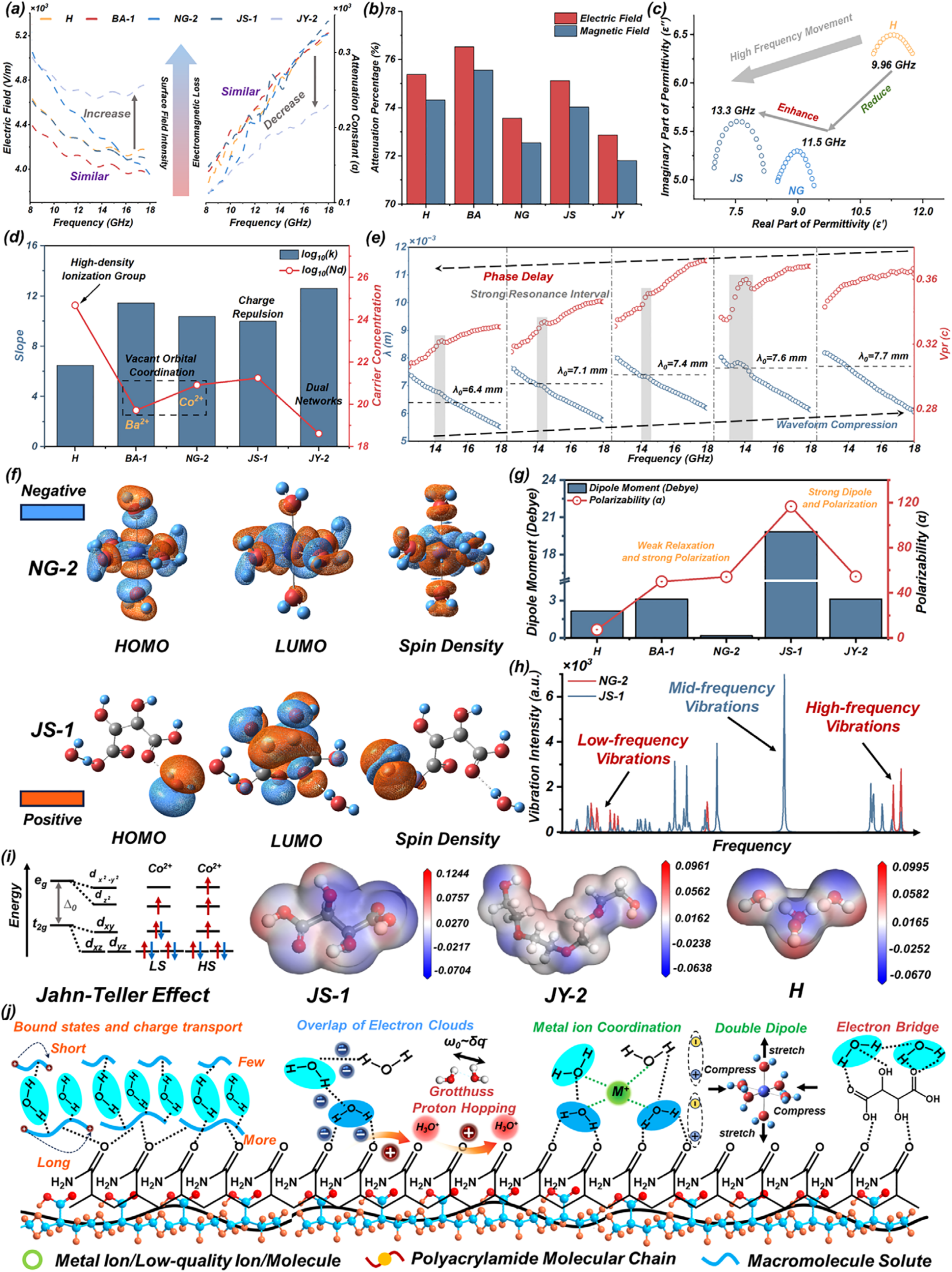
(a–c) The electric field, attenuation constant, and percentage and Cole–Cole plot of hydrogels. (d) The Mott–Schottky slope and logarithmic carrier concentration. (e) The Wavelength and traveling‐wave velocity of electromagnetic waves at 12.4–18.0 GHz in the hydrogels. (f) The HOMO, LUMO, and spin density of NG‐2 and JS‐1 with coordinated water molecules. (g) The dipole moment and polarizability of hydrogels. (h) The vibrational modes of NG‐2 and JS‐1 at different frequencies. (i) The Jahn–Teller effect of Co^2+^ and charge distributions of H, JS‐1, and JY‐2. (j) The electromagnetic wave loss mechanisms in pure hydrogels, inorganic salt gels, and organic molecule gels.

Finally, distinct dielectric‐regulation mechanisms govern the four hydrogel systems. In pure hydrogels, long‐range charge transport primarily follows the Grotthuss mechanism along hydrogen‐bonded pathways formed by polymer‐confined water, with network architecture and water distribution determining conduction loss. Meanwhile, hydrogen bonding between water molecules and polymer pendant groups induces local charge redistribution through electron‐cloud distortion, generating dipolar polarization sites. In contrast, transition metal cations coordinate with water molecules through interactions involving oxygen lone pairs, disrupting the original hydrogen‐bond network and restricting water mobility. The resulting metal‐water complexes become anchored within the polymer network, introducing additional dipolar polarization centers while suppressing proton transport. Unlike metal cations, ionizable organic molecules regulate dielectric behavior through their dissociated species and interactions with polymer pendant groups. Their electronegative functional groups can establish molecular bridges that reorganize proton‐hopping pathways, while H^+^‐donating species can induce protonation and generate additional polarization centers. By comparison, non‐ionizable soluble organics primarily restructure the confined water phase through noncovalent interactions rather than metal‐water coordination or proton donation. They partition continuous water domains and reorganize interchain spaces, thereby modifying charge‐transport pathways and associated polarization responses. These distinct mechanisms enable solute‐specific regulation of conduction and polarization losses, providing a basis for broadband microwave absorption (Figure 5j). Therefore, compared with composite hydrogels, the proposed system exhibits unique performance advantages and enhanced tunability (Figure S54 and Table S2).

### 2.5 The Mechanical Properties and Functionalization of Gels

In practical applications, editability, environmental adaptability, physical stability, and mechanical properties are all essential characteristics for electromagnetically compatible devices. Particularly for flexible wearable devices and electronic skins for intelligent robots, in addition to electromagnetic protection in specific frequency bands, high stretchability, compressibility, thermal stability, and environmental stability are also required. First, the electromagnetic parameters of the solute‐regulated hydrogel span a wide range. By modulating the polarization and conduction properties, the dielectric constant can be tuned from 3 to 15, fully covering the optimal matching region constrained by the Kramers–Kronig relations and Maxwell's equations, demonstrating strong editability of the dielectric properties (Figure 6a) [54, 55, 56]. Subsequently, using the CST antenna module, we adopted a spatial two‐port waveguide to simulate dual‐channel electromagnetic signal emission and statistically evaluated the spatial power loss of the gel in a unidirectional electromagnetic environment within an enclosed space. The gel exhibited a high‐power enhancement at high frequencies, with NG‐2 achieving the highest spatial electromagnetic utilization, reaching an energy of 6.5 W (Figure 6b). Considering the practical requirements of electronic skins and flexible wearables, we evaluated the gels’ stretchability, electromagnetic radiation attenuation, and load‐bearing capacity. Under the same tensile force, NG‐2 (175%) and JS‐1 (160%) exhibited greater stretchability than H (150%) (Figure 6c). When placed near a continuously charging mobile phone motherboard for 60 s, the gels reduced the detected electric‐field radiation level to 19.53% for H, 13.37% for NG‐2, and 14.44% for JS‐1, demonstrating effective electromagnetic radiation attenuation (Figure 6d,e). Tensile tests further confirmed solute‐induced mechanical enhancement, with BA‐1 achieving an elongation exceeding 400%. Under a 2.5 kg load, H failed to lift the weight, whereas NG‐2 and JS‐1 sustained it for over 30 s without fracture, confirming their improved load‐bearing capacity (Figure 6f,g). Considering the many influences of the actual environment, H, NG‐2, and JS‐1 were subjected to a 30‐day natural environment exposure test, during which the ambient temperature ranged from 15°C to 35°C. The results showed that the samples after 30 days exhibited no obvious deterioration, shrinkage, or corrosion, further demonstrating the environmental tolerance of this hydrogel polymer system (Figure 6h). Furthermore, considering the potential high‐temperature environments of processors and motherboard components, the gels were heated at 100°C for 5 min (Figure S55). The average surface temperature rise did not exceed 10°C and no obvious melting was observed, further indicating that specific solute‐modified hydrogels can maintain short‐term tolerance in high‐temperature environments (Figure 6i). In summary, solute‐tuned hydrogels, as electromagnetically compatible devices, provide a promising electromagnetic protection strategy for future flexible wearable devices and electronic skins for AI robots.

**FIGURE 6:**
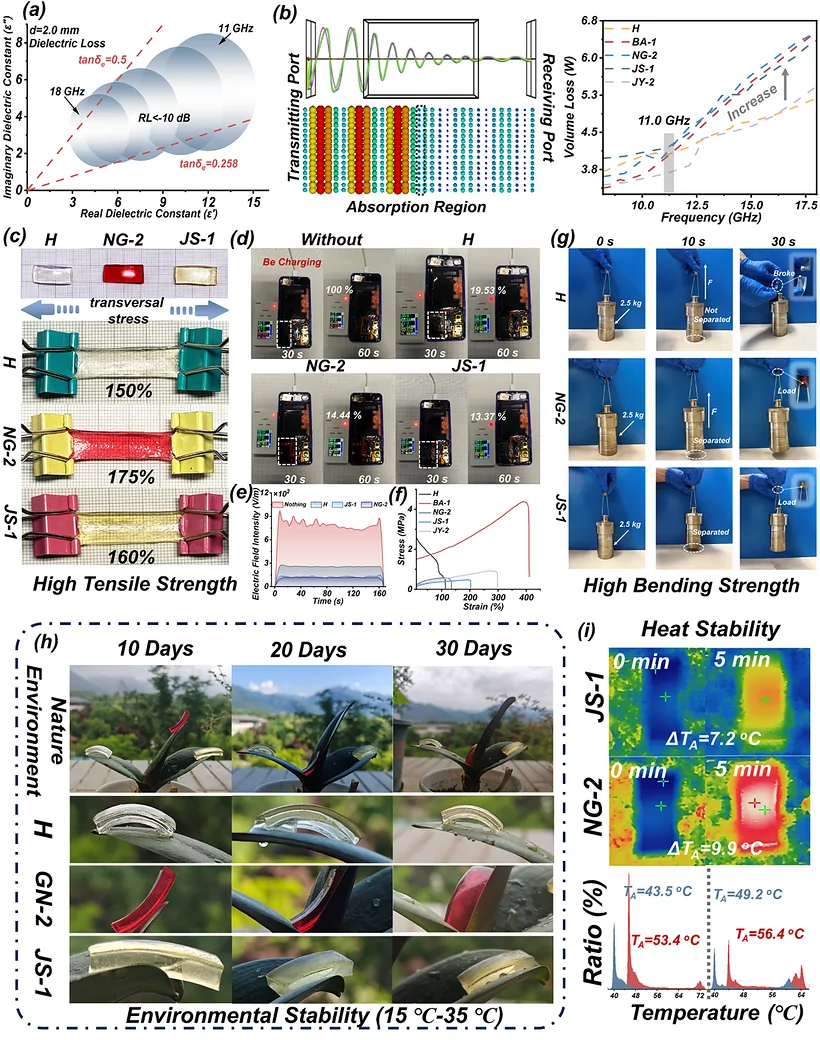
(a) The dynamic convergence range of the optimal solution set limited by the Maxwell equation of electromagnetic parameters (for effective reflection loss RL←10 dB), with a thickness of 2.0 mm and frequency from 11.0 to 18.0 GHz. (b) The volume loss of hydrogels. (c–f) The photograph, transverse stretching demonstration, radiation test of the patch applied to a mobile phone, and tensile test of H, NG‐2, and JS‐1. (g) The load‐bearing capacity test of H, NG‐2, and JS‐1 from 0 to 30 s with 2.5 kg. (h) The 30‐day environmental stability demonstration of hydrogels. (i) The thermal stability test and surface mean temperature statistics of NG‐2 and JS‐1.

## 3 Conclusion

In summary, we developed a solute‐mediated dielectric regulation strategy in AA/AM hydrogels by independently introducing hydrogen bonding, metal‐ion hydration, and protonation to explore their roles in electromagnetic response. The results demonstrate that hydrogen bonding and metal‐ion coordination suppress proton hopping and generate additional dipolar polarization units, whereas protonation simultaneously regulates water conduction and hydrogen‐bond‐induced polarization, thereby enabling a controllable balance between conduction and polarization losses. In particular, Co^2+^ coordination induced by *d*‐orbital interactions suppressed conduction loss by 2.25 while enhancing polarization loss by 1.02, whereas tartaric‐acid‐induced protonation simultaneously increased conduction and polarization losses by 0.61 and 0.95, respectively. By coupling Co^2^^+^‐induced hydrated ions with backbone hydrogen bonding and tartaric‐acid‐induced protonation with polar‐group hydrogen bonding, ultrawide EAB of 7.1 GHz was achieved at thicknesses of 1.88 and 1.85 mm, respectively. These findings establish a clear structure‐interaction‐dielectric relationship for water‐mediated noncovalent interactions and provide a general strategy for rationally tuning hydrogel dielectric properties toward broadband microwave absorption and flexible electromagnetic devices.

## Author Contributions

**Limin Zhang**, **Hongjing Wu**, and **Wei Zhou** conceived and supervised the research. **Zijing Li** and **Xiangdong Wang** performed the experiments and simulations and analyzed the data. Zijing Li wrote the paper. **Shengchong Hui** provided key simulation data. **Gaoxu Zhang** and **Jiaqi Wang** provided key characterization data.

## Conflicts of Interest

The authors declare no conflicts of interest.

## Ethics Statement

The authors declare no competing interests.

## Supporting information




**Supporting File**: advs78744‐sup‐0001‐SuppMat.docx.

## Data Availability

The data that support the findings of this study are available from the corresponding author upon reasonable request.
