# Supplementary material for: Solute‐Induced Modulation of Hydrogen Bonding, Ion Hydration, and Protonation in Hydrogels for Enhanced Microwave Absorption
Source: Adv Sci (Weinh). 2026 Nov 9:e78744. Online ahead of print. doi: 10.1002/advs.78744 (PMC13748890; doi:10.1002/advs.78744)
Supplement: Supplementary file 1 [file ADVS-9999-e78744-s001.docx]

**Solute Induced Modulation of Hydrogen Bonding, Ion** **Hydration, and Protonation in Hydrogels for Enhanced Microwave Absorption**

Zijing Li ^1,#^ , Xiangdong Wang ^1,#^, Shengchong Hui ^1^, Gaoxu Zhang ^1^, Jiaqi Wang ^1^, Wei Zhou^3,*^, Hongjing Wu ^1,4*^, Limin Zhang ^1,2*^

^1^ MOE Key Laboratory of Material Physics and Chemistry under Extraordinary Conditions, School of Physical Science and Technology, Northwestern Polytechnical University, Xi’an 710072, P.R. China;

^2^ Shenzhen Research Institute of Northwestern Polytechnical University, Sanhang Science &Technology Buliding, No. 45th, Gaoxin South 9th Road, Nanshan District, Shenzhen City, 518057, Guangdong, China;

^3^ Hunan Key Laboratory of Applied Environmental Photocatalysis, Changsha University, Changsha

410022, China;

^4^ State Key Laboratory of Powder Metallurgy, Central South University, Changsha, 410083, China

^*^ Corresponding authors: zhouwei_csu@163.com (W. Zhou); [wuhongjing@nwpu.edu.cn](mailto:wuhongjing@nwpu.edu.cn) (H. Wu); liminzhang@nwpu.edu.cn (L. Zhang)

^#^ These authors contributed equally to this work.

**Method**

**Materials**

Acrylic acid (C_3_H_4_O_2_), acrylamide (C_3_H_5_NO), N,N'-methylene diacrylamide (MBA, C_7_H_10_N_2_O_2_), photoinitiator, deionized water, absolute ethanol, various soluble metal salts and organic solvents were purchased from Shanghai Macklin Biochemical Co., Ltd (China). All the chemicals were analytically pure without further purification.

**Preparation of CN system**

In this study, hydrogel samples with different solutes were prepared by UV curing at room temperature. First, water-soluble metal salts, acids, or organic molecules at various concentrations were dissolved in 7 mL of deionized water under magnetic stirring for 60 min until completely dissolved, forming stock solutions. Subsequently, 5.0 g of acrylic acid (AA), 4.0 g of acrylamide (AM), and 0.09 g of N,N'-methylenebisacrylamide (MBA) were mixed into the prepared solution and magnetically stirred for 30 min until the solids were fully dissolved. Then, 0.01 g of photoinitiator was added, and the mixture was stirred for an additional 5 to 10 min. The solution was placed in a vacuum drying oven to remove air bubbles introduced during stirring. Finally, the degassed solution was cast into PTFE molds and cured under a 365 nm UV lamp for 12 h, yielding hydrogel samples with different solute types and concentrations. For inorganic metal salt solutions, a series of concentrations was designed according to the valence of the cations to maintain equivalent ionic charge concentrations. Specifically, the concentration gradients were set at 0.5, 1.0, and 1.5 for monovalent cations; 0.25, 0.5, and 0.75 for divalent cations; and 0.17, 0.33, and 0.5 for trivalent cations. For short-chain organic molecules, concentration gradients of 0.5, 1.0, and 1.5 were adopted, provided that all concentrations remained below the respective solubility limits. For organic polymers exhibiting relatively large variations in mass fraction, samples were prepared using a mass ratio of 1:2:3.

**Characterization**

The elemental composition, functional groups, and chemical bonding states of the products were characterized using X‑ray photoelectron spectroscopy (XPS, Kratos AXIS Ultra DLD, UK), Raman spectroscopy (HORIBA LabRAM HR Evolution, Japan), and Fourier‑transform infrared spectroscopy (FTIR, Thermo Scientific Nicolet iS20, USA). The microstructure, morphology, cross‑sectional features, and elemental distribution were examined with a scanning electron microscope (SEM, ZEISS Sigma 360, Germany) equipped with an energy‑dispersive X‑ray spectrometer (EDS). Prior to SEM observation, the samples were pre‑frozen in a cold trap at -50 °C. Electrochemical impedance spectroscopy (EIS) and Mott-Schottky analysis were performed on a CHI 660D electrochemical workstation. For impedance measurements, a platinum foil, Ag/AgCl, and a 3 M KOH aqueous solution served as the working electrode, reference electrode, and electrolyte, respectively. Mott–Schottky analysis employed a 1 M Na_2_SO_4_ aqueous electrolyte. The mechanical properties and thermal management characteristics of the hydrogels were evaluated using an electronic universal testing machine (CMT6103) and an infrared thermal imaging camera.

**Electromagnetic measurement and analysis**

The vector network (VNA, Anritsu MS46322B) was used to investigate the electromagnetic material parameters of samples at frequency of 8.2 GHz to18.0 GHz. Before the measurements, the sample dimensions were ensured to meet the requirements for the X‑band (22.86 × 10.16 mm) and Ku‑band (15.80 × 7.90 mm) tests. The *RL* value is given according to transmission line theory as follows:

$RL=20\cdot\log\left|\frac{Z_{in}-Z_{0}}{Z_{in}+Z_{0}}\right|$ (1)

$Z_{in}=Z_{0}\cdot\sqrt{\frac{\mu_{r}}{\varepsilon_{r}}}\tanh(j\frac{2\pi fd}{c}\sqrt{\mu_{r}\varepsilon_{r}})$ (2)

Where, *Z_0_*, *Z_in_*, *d* and *c* denote the impedance of free space, the input impedance, the absorber thickness, and the speed of light in vacuum, respectively.

When an electromagnetic wave enters a material, its propagation velocity and attenuation differ from those in vacuum. The propagation constant is expressed as:

$\gamma=\alpha+j\beta=j\omega\sqrt{\varepsilon\mu}$ (3)

$\alpha=\frac{\sqrt{2}\pi f}{c}\sqrt{\mu'\varepsilon'}\sqrt{\tan\delta_{e}\tan\delta_{m}-1+\sqrt{(1+\tan^{2}\delta_{e})(1+\tan^{2}\delta_{m})}}$ (4)

$\beta=\frac{\sqrt{2}\pi f}{c}\sqrt{\mu'\varepsilon'}\sqrt{1-\tan\delta_{e}\tan\delta_{m}+\sqrt{(1+\tan^{2}\delta_{e})(1+\tan^{2}\delta_{m})}}$ (5)

$\tan\delta_{e}=\frac{\varepsilon''}{\varepsilon'}$ (6)

$\tan\delta_{m}=\frac{\mu''}{\mu'}$ (7)

*α* is the attenuation constant of the transmission line, representing the factor by which the amplitude of an electromagnetic wave decays by *e^-α^* per unit length. *β* is the phase shift constant of the transmission line, indicating the radian measure of phase lag per unit length. *tanδₑ* is the dielectric loss tangent, and *tanδₘ* is the magnetic loss tangent.

To better discern the relationship between variations in electromagnetic parameters and the reflection of electromagnetic waves, equation is approximated using a trigonometric approach and simplified into a *Δ* function. The smaller the absolute value of *Δ*, the closer the system approaches perfect impedance matching:

$\frac{Z}{Z_{0}}=\sqrt{\frac{\mu_{r}}{\varepsilon_{r}}}\tanh(\gamma d)=1$ (8)

$\left|\Delta\right|=\left|\sinh^{2}(Kfd)-M\right|=0$ (9)

$K=\frac{4\pi\sqrt{\mu'\varepsilon'}}{c\cos\delta_{e}\cos\delta_{m}}\sin\frac{\delta_{e}+\delta_{m}}{2}$ (10)

$M=\frac{4\mu'\cos\delta_{e}\varepsilon'\cos\delta_{m}}{\left(\mu'\cos\delta_{e}-\varepsilon'\cos\delta_{m}\right)^{2}+\left[\tan(\frac{\delta_{m}}{2}-\frac{\delta_{e}}{2})\right]^{2}(\mu'\cos\delta_{e}+\varepsilon'\cos\delta_{m})}$ (11)

The polarization relaxation phenomenon induced by an increasing frequency of the alternating electric field is directly reflected in the complex permittivity of a material. Moreover, the relaxation behavior of dipole polarization can be further described by the Debye relaxation theory. According to this theory, the orientation of polar molecules under an alternating current is influenced by both thermal motion and intermolecular interactions. The effect of intermolecular interactions can be represented using a viscous damping analogy, in which molecules are treated as spheres moving in a continuous medium with macroscopic viscosity. The dispersion and absorption effects observed in a sufficient number of liquids and dielectrics can be expressed by the following formula:

$\varepsilon''=\varepsilon''_{p}+\varepsilon''_{c}=\frac{2\pi f\tau(\varepsilon_{s}-\varepsilon_{\infty})}{1+(2\pi f)^{2}\tau^{2}}+\frac{\sigma}{2\pi f\varepsilon_{0}}$ (12)

(13)

$\left(\varepsilon'-\frac{\left(\varepsilon_{s}-\varepsilon_{\infty}\right)}{2}\right)+\left(\varepsilon''\right)^{2}=\frac{(\varepsilon_{s}-\varepsilon_{\infty})^{2}}{2}$ (14)

*ε_0_* and *ε_∞_* are the permittivities at frequencies approaching zero and infinity, respectively; *τ_0_* is the generalized relaxation time, the angular frequency *ω=2πf*. The equation (14) requires that the trajectory of the complex permittivity on the complex plane be an arc, with its endpoints on the real axis and its centre below the real axis.

$d_{m}=\frac{2n+1}{4}\frac{c}{f(\left|\mu_{r}\right|\left|\varepsilon_{r}\right|)^{\frac{1}{2}}}$ (15)

Upon entering a material, the intrinsic mechanisms governing electromagnetic wave loss vary significantly among different materials. In electromagnetism, the energy of an electromagnetic field is conventionally described by the energy density *ω* and the energy flux density *S* (the Poynting vector). The conservation of energy in an electromagnetic field can then be expressed as:

$\frac{\partial\omega}{\partial t}+\nabla\cdot S+f\cdot v=0$ (16)

$J\cdot E=J_{f}\cdot E+J_{p}\cdot E+J_{m}\cdot E$ (17)

where *v* is the velocity of charge motion, *f* is the Lorentz force per unit volume of the medium, *J_f_* is the free charge density, *J_p_* is the polarization current density, *P* is the polarization intensity representing the electric dipole moment per unit volume of the medium, *J_m_* is the magnetization current density, and *M* is the magnetization intensity representing the magnetic dipole moment per unit volume of the medium.

The three current densities in the equation (17) correspond to three distinct forms of electromagnetic energy loss within the medium. The first term on the right‑hand side represents the work performed by the electromagnetic field on the free electrons in the medium, converting electromagnetic energy into kinetic energy of the free electrons. However, owing to the resistive nature of the medium, Joule’s law dictates that this energy conversion ultimately results in Joule heat (a process equally applicable to macroscopic currents induced by the varying electromagnetic field). The second term on the right‑hand side describes the polarization of the medium by the electric field, signifying the conversion of electromagnetic field energy into polarization energy stored in the medium. The third term corresponds to the magnetization of the medium by the electromagnetic field, through which the medium gains energy; during this process, the magnetization effect induces a transformation of electromagnetic field energy into other forms of energy.

The Kramers-Kronig (K-K) relation unifies the frequency domain with causal variations. As a Hilbert transform that connects the real and imaginary parts of the permittivity, its origin lies in the causality constraint of linear systems. For any linear medium that obeys causality, an interlocking relationship exists between the real part *ε′(ω)* and the imaginary part *ε″(ω)* in the frequency domain. For instance, a dispersive feature in the real part, such as a steep decline, is inevitably accompanied by a peak in the imaginary part at a specific frequency.

(18)

$\varepsilon''(\omega)=-\frac{2\omega}{\pi}P\int_{0}^{\infty} \frac{\varepsilon'(\omega')-\varepsilon_{\infty}}{\omega'^{2}-\omega^{2}}d\omega'$ (19)

$\int_{0}^{\infty} \omega\varepsilon''(\omega)d\omega=\frac{\pi}{2}\omega_{p}^{2}$ (20)

where *P* denotes the Cauchy principal value integral and *ε_∞_* is the high‑frequency limit permittivity. The sum rule derived from the K-K relation imposes a constraint on the total absorption of a material. Equation (20) demonstrates that the absorption capability of a material is globally limited by its free carrier concentration. An attempt to achieve extremely high loss within a single narrow band would inevitably induce dispersion anomalies in other frequency ranges, thereby deviating from linear behavior.

Equivalent circuit model for the Smith chart: The conductive process is represented by a parallel RC branch, whereas polarization relaxation is described by one or more series RC branches connected in parallel with a capacitive branch representing the high-frequency dielectric response. The combinations of resistance and capacitance values determine the overall magnitudes of conductive and polarization losses, thereby modulating the trajectory of the impedance response on the Smith chart.

The K-K relation and Maxwell’s equations must form a dual constraint in the design of wave materials. The K-K relation imposes a global causality constraint in the frequency domain, ensuring the self‑consistency between the real and imaginary parts of the permittivity. In the GHz regime, any design that enhances the imaginary part for narrowband absorption inevitably induces a dispersive variation of the real part over a broader frequency range. Maxwell’s equations, in turn, provide the propagation equations for electromagnetic waves in materials and the interface matching conditions, which determine the fundamental criteria that the macroscopic electromagnetic effects of these material parameters in both space and frequency must satisfy. The imaginary part of the permittivity directly determines the penetration depth of electromagnetic waves in the medium:

$\nabla\cdot\left[\varepsilon_{0}\varepsilon(\omega)E\right]=\rho_{f}$ (21)

$\nabla\cdot\left[\mu_{0}\mu(\omega)H\right]=0$ (22)

$\nabla\times H=-i\omega\left[\varepsilon_{0}\varepsilon(\omega)E\right]$ (23)

$\nabla\times E=i\omega\left[\mu_{0}\mu(\omega)H\right]$ (24)

$E(z)=E_{0}e^{-\alpha z}e^{i(\beta z-\omega t)}$ (25)

where *D* is the electric displacement field, *ρ_f_* is the free charge density, *H* is the magnetic field strength, *B* is the magnetic flux density, and E is the electric field strength.

**Electromagnetic simulation and image identification methods**

A coupled model was established in COMSOL Multiphysics using the Electric Currents (ec) interface in the AC/DC Module and the Electromagnetic Waves, Frequency Domain (emw) interface in the RF module. To cover the absorption frequency range of the optimal samples, the relative permittivity and dielectric loss tangent were selected as variables. The frequency domain was set to 11.0-18.0 GHz with an interval of 1.0 GHz, and a physics-controlled extremely fine mesh was employed. The stationary solver was configured to use the MUMPS solver, with a pivot threshold of 0.01 and a tolerance of 10^-8^. A fully coupled approach was adopted for the calculations. The iterative solver was set to BiCGStab, with a maximum of 10,000 iterations. The final results included the electric potential and the x-, y-, and z-components of the electric field.

The wavelength and propagation velocity of a traveling wave in a medium are described by a coupled model involving the Helmholtz equation and the Poynting vector equation.

Helmholtz equations derived from the source-free Maxwell equations:

$\nabla^{2}E+\omega^{2}\mu\varepsilon E=0$ (26)

$\nabla^{2}H+\omega^{2}\mu\varepsilon H=0$ (27)

$E=E_{0}e^{i\omega t-\gamma_{E}z}$ (28)

$H=H_{0}e^{i\omega t-\gamma_{H}z}$ (29)

Here, E and H denote the electric and magnetic field; ε and μ represent the complex permittivity and permeability; ω and t denote the angular frequency and time; and γ is the propagation constant.

Time-averaged power loss density derived from the Poynting vector:

$\mathrm{Re}(\nabla\bullet S)=\frac{\omega}{2}(\varepsilon''\left|E\right|^{2}+\mu''\left|H\right|^{2})$ (30)

$\mathrm{Im}(\nabla\bullet S)=\frac{\omega}{2}(\varepsilon'\left|E\right|^{2}+\mu'\left|H\right|^{2})$ (31)

By combining equations (26)-(31), the wavelength and relative propagation velocity of electromagnetic traveling waves in a lossy medium can be derived as follows:

$\gamma=\frac{2i\pi f}{c}\bullet\sqrt{\varepsilon'\mu'-i\varepsilon'\mu''-i\varepsilon''\mu'-\varepsilon''\mu''}$ (32)

$\beta=\mathrm{Im}\left(\gamma\right)$ (33)

$\nu=\frac{2\pi f}{\beta c}$ (34)

The coordination models of organic molecules, metal cations, and water molecules were first optimized using the Forcite GeomOpt module to identify the lowest-energy configurations. The optimized structures were then used as input models for density functional theory (DFT) calculations in Gaussian, with the bond lengths, bond angles, and hydrogen-bond positions constrained. The B3LYP hybrid functional was employed to describe exchange-correlation interactions, together with the def2SVP basis set, to obtain the molecular orbital energy levels, electron density distributions, and relevant dielectric properties. The initial self-consistent field (SCF) settings were configured using the Mix algorithm, with a convergence criterion of 10^-8^ and a maximum of 500 iterations.

For the polarizability (α), the isotropic average of the polarizability tensor was calculated as follows:

$\bar{\alpha}=\frac{\alpha_{xx}+\alpha_{yy}+\alpha_{zz}}{3}$ (35)

The magnitude of the dipole moment is given by:

$\left|\mu\right|=\sqrt{\mu_{x}^{2}+\mu_{y}^{2}+\mu_{z}^{2}}$ (36)

The dipole moment is typically expressed in Debye, whereas the components of the polarizability tensor are generally reported in atomic units (a.u.).

The surface temperature distribution plots were generated using an image-processing program. First, the RGB values (0-255) of each pixel in the acquired thermal images were processed through grayscale conversion and channel separation. Each pixel was then assigned a grayscale value, which was mapped to the corresponding temperature range to obtain the temperature distribution curve. Finally, the temperature scale displayed on the right side of the image was used as a reference to digitize the pixel values and fit the corresponding temperature values.

The electric-field distributions and electromagnetic field loss percentages were simulated using a waveguide model. The simulations were performed in CST Studio Suite using the Antennas workflow in the Microwave & RF/Optical module, with the time-domain solver configured for the waveguide model. Single-frequency simulations were conducted at the optimal impedance-matching thickness. Because the material structure itself is polarization-insensitive, the antenna model employed two waveguide ports, S1 and S2, for signal transmission and reception. The transmitted signal was vertically polarized, and the separation between the two ports was set to greater than one wavelength. Signals at the designated boundary surfaces were truncated by imposing (E_t_=0) and (H_t_=0), ensuring the spatial propagation of electromagnetic waves along the normal direction. The background level in the simulation environment was set to -80 dB, substantially lower than the minimum reflection loss values of all the materials, ensuring that the influence of environmental background noise could be considered negligible.

**Table S1** Nomenclature of gels with different solutes

| No. | Samples | Sample code | No. | Samples | Sample code |
| --- | --- | --- | --- | --- | --- |
| 1 | H_2_O | H | 17 | KBr | KB |
| 2 | NaCl | Na | 18 | BaCl_2_ | BA |
| 3 | NH_4_Cl | NH | 19 | C_6_H_5_Na_3_O_7_ | NM |
| 4 | NaNO_3_ | XN | 20 | C_6_H_9_CeO_6_ | CSH |
| 5 | Na_2_SO_4_ | SN | 21 | LaCl_3_ | LA |
| 6 | NaOH | HN | 22 | CH_3_COOH | CS |
| 7 | SnCl_2_ | Sn | 23 | CeCl_3_ | LS |
| 8 | (CH_3_COO)_2_Zn | CZ | 24 | HNO_3_ | XS |
| 9 | C_7_H_5_O_3_Na | SY | 25 | HCl | Y |
| 10 | NH_4_H_2_PO_4_ | PN | 26 | (CH_3_COO)_2_Ba | YB |
| 11 | La(NO_3_)_3_ | La | 27 | CH_3_COONa | YN |
| 12 | CoCl_2_ | GU | 28 | PVP(58000) | PVP-2 |
| 13 | ZnCl_2_ | ZN | 29 | C_6_H_12_O_6_ | PU |
| 14 | MnCl_2_ | MN | 30 | CO(NH_2_)_2_ | N |
| 15 | Ce(NO_3_)_3_ | NS | 31 | C_8_H_12_ClNO_2_ | YS |
| 16 | Co(NO_3_)_2_ | NG | 32 | C_4_H_6_N_2_ | RJ |
| No. | Samples | Sample code | No. | Samples | Sample code |
| 33 | C_2_H_5_NO_2_ | GA | 39 | C_76_H_52_O_46_ | DN |
| 34 | C_4_H_6_O_6_ | JS | 40 | C_24_H_46_O_12_ | JB |
| 35 | C_5_H_14_ClNO | LD | 41 | (C_3_H_5_NO)_n_ | BX |
| 36 | C_6_H_8_O_7_ | NN | 42 | PEG | JY |
| 37 | CH_6_ClN_3_ | YG | 43 | [C_6_H_10_O_5_]_n_ | PJ |
| 38 | PVP(10000) | PVP-1 |  |  |  |

The actual samples were named X‑1, X‑2, and X‑3 according to the solute concentration prepared, from low to high. For example, hydrogels prepared with 0.5 mol/L, 1.0 mol/L, and 1.5 mol/L NaCl solutions were designated Na‑1, Na‑2, and Na‑3, respectively.

**Supplementary Fig. 1** The C 1s, O 1s and N 1s XPS spectra of YB-3, LA-1, CS-3 and XN-1.

**Supplementary Fig. 2** The C 1s, O 1s and N 1s XPS spectra of BA-3, Y-3, YB-3 and La-1.

**Supplementary Fig. 3** The C 1s, O 1s and N 1s XPS spectra of XS-3, JC-2, JS-3 and PJ-3.

**Supplementary Fig. 4** The C 1s, O 1s and N 1s XPS spectra of BX-1, PVP-1-1, PVP-2-1 and Y-1.

**Supplementary Fig. 5** The C 1s, O 1s and N 1s XPS spectra of JY-1, JY-3, GA-1 and DN-1.

**Supplementary Fig. 6** The carrier concentration of the positive slope of tangent.

**Supplementary Fig. 7** The carrier concentration of the negative slope of tangent.

**Supplementary Fig. 8** The fourier transform infrared spectroscopy of inorganic and organic systems.

**Supplementary Fig. 9** The electrochemical impedance spectroscopy of inorganic and organic systems.

**Supplementary Fig. 10** The calibration parameters of PTFE for the X and Ku bands.

**Supplementary Fig. 11** The electromagnetic parameter and 2D reflection loss (*RL*) mappings of pure hydrogel, 0.5 mol/L, 1.0 mol/L and1.5 mol/L NaCl hydrogels.

**Supplementary Fig. 12** The electromagnetic parameter and 2D reflection loss (*RL*) mappings of 0.5 mol/L, 1.0 mol/L and1.5 mol/L NH_4_Cl hydrogels.

**Supplementary Fig. 13** The electromagnetic parameter and 2D reflection loss (*RL*) mappings of 0.5 mol/L, 1.0 mol/L and1.5 mol/L NaNO_3_ hydrogels.

**Supplementary Fig. 14** The electromagnetic parameter and 2D reflection loss (*RL*) mappings of 0.25 mol/L, 0.5 mol/L and 0.75 mol/L Na_2_SO_4_ hydrogels.

**Supplementary Fig. 15** The electromagnetic parameter and 2D reflection loss (*RL*) mappings of 0.5 mol/L, 1.0 mol/L and 1.5 mol/L NaOH hydrogels.

**Supplementary Fig. 16** The electromagnetic parameter and 2D reflection loss (*RL*) mappings of 0.5 mol/L, 1.0 mol/L and 1.5 mol/L SnCl_2_ hydrogels.

**Supplementary Fig. 17** The electromagnetic parameter and 2D reflection loss (*RL*) mappings of 0.25 mol/L, 0.5 mol/L and 0.75 mol/L C_4_H_6_O_4_Zn hydrogels.

**Supplementary Fig. 18** The electromagnetic parameter and 2D reflection loss (*RL*) mappings of 0.5 mol/L, 1.0 mol/L and 1.5 mol/L C_7_H_5_O_3_Na hydrogels.

**Supplementary Fig. 19** The electromagnetic parameter and 2D reflection loss (*RL*) mappings of 0.5 mol/L, 1.0 mol/L and 1.5 mol/L NH_4_H_2_PO_4_ hydrogels.

**Supplementary Fig. 20** The electromagnetic parameter and 2D reflection loss (*RL*) mappings of 0.17 mol/L, 0.33 mol/L and 0.25 mol/L La(NO_3_)_3_ hydrogels.

**Supplementary Fig. 21** The electromagnetic parameter and 2D reflection loss (*RL*) mappings of 0.25 mol/L, 0.5 mol/L and 0.75 mol/L CoCl_2_ hydrogels.

**Supplementary Fig. 22** The electromagnetic parameter and 2D reflection loss (*RL*) mappings of 0.25 mol/L, 0.5 mol/L and 0.75 mol/L ZnCl_2_ hydrogels.

**Supplementary Fig. 23** The electromagnetic parameter and 2D reflection loss (*RL*) mappings of 0.25 mol/L, 0.5 mol/L and 0.75 mol/L MnCl_2_ hydrogels.

**Supplementary Fig. 24** The electromagnetic parameter and 2D reflection loss (*RL*) mappings of 0.17 mol/L, 0.25 mol/L and 0.33 mol/L Ce(NO_3_)_3_ hydrogels.

**Supplementary Fig. 25** The electromagnetic parameter and 2D reflection loss (*RL*) mappings of 0.5 mol/L, 1.0 mol/L and 1.5 mol/L KBr hydrogels.

**Supplementary Fig. 26** The electromagnetic parameter and 2D reflection loss (*RL*) mappings of 0.25 mol/L, 0.5 mol/L and 0.75 mol/L Co(NO_3_)_2_ hydrogels.

**Supplementary Fig. 27** The electromagnetic parameter and 2D reflection loss (*RL*) mappings of 0.25 mol/L, 0.5 mol/L and 0.75 mol/L BaCl_2_ hydrogels.

**Supplementary Fig. 28** The electromagnetic parameter and 2D reflection loss (*RL*) mappings of 0.5 mol/L, 1.0 mol/L and 1.5 mol/L C_6_H_5_Na_3_O_7_ hydrogels.

**Supplementary Fig. 29** The electromagnetic parameter and 2D reflection loss (*RL*) mappings of 0.5 mol/L, 1.0 mol/L and 1.5 mol/L Ce(C_2_H_3_O_2_)_3_ hydrogels.

**Supplementary Fig. 30** The electromagnetic parameter and 2D reflection loss (*RL*) mappings of 0.17 mol/L, 0.33 mol/L and 0.5 mol/L LaCl_3_ hydrogels.

**Supplementary Fig. 31** The electromagnetic parameter and 2D reflection loss (*RL*) mappings of 0.17 mol/L, 0.33 mol/L and 0.5 mol/L CeCl_3_ hydrogels.

**Supplementary Fig. 32** The electromagnetic parameter and 2D reflection loss (*RL*) mappings of 0.25 mol/L, 0.5 mol/L and 0.75 mol/L Ba(CH_3_COO)_2_ hydrogels.

**Supplementary Fig. 33** The electromagnetic parameter and 2D reflection loss (*RL*) mappings of 0.5 mol/L, 1.0 mol/L and 1.5 mol/L NaCH_3_COO hydrogels.

**Supplementary Fig. 34** The electric field modulus of NG-2 from 12.0 GHz to 17.0 GHz.

**Supplementary Fig. 35** The electromagnetic parameter and 2D reflection loss (*RL*) mappings of 1.0 mol/L, 2.0 mol/L and 3.0 mol/L CH_4_N_2_O hydrogels.

**Supplementary Fig. 36** The electromagnetic parameter and 2D reflection loss (*RL*) mappings of 1.0 mol/L, 2.0 mol/L and 3.0 mol/L C_8_H_12_ClNO_2_ hydrogels.

**Supplementary Fig. 37** The electromagnetic parameter and 2D reflection loss (*RL*) mappings of 1.0 mol/L, 2.0 mol/L and 3.0 mol/L C_4_H_6_N_2_ hydrogels.

**Supplementary Fig. 38** The electromagnetic parameter and 2D reflection loss (*RL*) mappings of 1.0 mol/L, 2.0 mol/L and 3.0 mol/L C_2_H_5_NO_2_ hydrogels.

**Supplementary Fig. 39** The electromagnetic parameter and 2D reflection loss (*RL*) mappings of 1.0 mol/L, 2.0 mol/L and 3.0 mol/L C_4_H_6_O_6_ hydrogels.

**Supplementary Fig. 40** The electromagnetic parameter and 2D reflection loss (*RL*) mappings of 1.0 mol/L, 2.0 mol/L and 3.0 mol/L C_5_H_14_ClNO hydrogels.

**Supplementary Fig. 41** The electromagnetic parameter and 2D reflection loss (*RL*) mappings of 1.0 mol/L, 2.0 mol/L and 3.0 mol/L C_6_H_8_O_7_ hydrogels.

**Supplementary Fig. 42** The electromagnetic parameter and 2D reflection loss (*RL*) mappings of 1.0 mol/L, 2.0 mol/L and 3.0 mol/L CH_6_ClN_3_ hydrogels.

**Supplementary Fig. 43** The electromagnetic parameter and 2D reflection loss (*RL*) mappings of 1.0 g, 2.0 g and 3.0 g PVP-10000 hydrogels.

**Supplementary Fig. 44** The electromagnetic parameter and 2D reflection loss (*RL*) mappings of 1.0 g, 2.0 g and 3.0 g PVP-58000 hydrogels.

**Supplementary Fig. 45** The electromagnetic parameter and 2D reflection loss (*RL*) mappings of 1.0 mol/L, 2.0 mol/L and 3.0 mol/L C_24_H_46_O_12_ hydrogels.

**Supplementary Fig. 46** The electromagnetic parameter and 2D reflection loss (*RL*) mappings of 1.0 mol/L, 2.0 mol/L and 3.0 mol/L C_76_H_52_O_46_ hydrogels.

**Supplementary Fig. 47** The electromagnetic parameter and 2D reflection loss (*RL*) mappings of 1.0 mol/L, 2.0 mol/L and 3.0 mol/L C_4_H_7_NO hydrogels.

**Supplementary Fig. 48** The electromagnetic parameter and 2D reflection loss (*RL*) mappings of 1.0 mol/L, 2.0 mol/L and 3.0 mol/L (C_3_H_5_NO)_n_ hydrogels.

**Supplementary Fig. 49** The electromagnetic parameter and 2D reflection loss (*RL*) mappings of 1.0 mol/L, 2.0 mol/L and 3.0 mol/L PEG hydrogels.

**Supplementary Fig. 50** The electromagnetic parameter and 2D reflection loss (*RL*) mappings of 1.0 mol/L, 2.0 mol/L and 3.0 mol/L (C_6_H_10_O_5_)_n_ hydrogels.

**Supplementary Fig. 51** The electromagnetic parameter and 2D reflection loss (*RL*) mappings of 1.0 mol/L, 2.0 mol/L and 3.0 mol/L HNO_3_ hydrogels.

**Supplementary Fig. 52** The electromagnetic parameter and 2D reflection loss (*RL*) mappings of 1.0 mol/L, 2.0 mol/L and 3.0 mol/L HCl hydrogels.

**Supplementary Fig. 53** The electromagnetic parameter and 2D reflection loss (*RL*) mappings of 1.0 mol/L, 2.0 mol/L and 3.0 mol/L C_6_H_12_O_6_ hydrogels.

**Supplementary Fig. 54** The EAB and thickness comparisons of NG-2 and JS-1 with other hydrogels and ionogels.

| Absorber | EAB (GHz) | | Matching  Thickness *d* (mm) | Refs. |
| --- | --- | --- | --- | --- |
| [DAC]5[Dy (NCS)8] gel | | 6.4 | 2.4 | [1] |
| RGO-Fe_3_O_4_ composite hydroge | | 5.3 | 2.0 | [2] |
| Flexible borate ester bonded hydrogels | | 5.6 | 2.1 | [3] |
| GO/Fe_3_O_4_ hydrogel | | 6.8 | 2.8 | [4] |
| A6G20T20-2 | | 4.2 | 2.9 | [5] |
| PP@M-LiCl hydrogel | | 4.2 | 0.9 | [6] |
| GO hydrogel | | 6.2 | 2.3 | [7] |
| MPAR-CVD | | 4.5 | 2.7 | [8] |
| NiFe_2_O_4_/CNTs organohydrogel | | 6.2 | 2.6 | [9] |
| Ni_x_Co_y_-C/polyacrylamide hydrogels | | 5.8 | 2.3 | [10] |
| Fe_2_O_3_@CNTs/PAM | | 3.4 | 1.7 | [11] |
| GO-HNT hydrogels | | 4.8 | 2.5 | [12] |
| PAM-5 | | 5.7 | 2.1 | [13] |
| NG-2 | | 7.1 | 1.9 | This Work |
| JS-1 | | 7.1 | 1.8 | This Work |

**Table S2** Comparison of this work with some hydrogels and ionogels in the literature.

**Supplementary Fig. 55** The temperature changes statistical chart of part hydrogels.

**Electromagnetic calculations and multi-scale simulations**

The computational framework for electromagnetic calculations, multiscale simulations, and machine learning was implemented in MATLAB to enable comprehensive characterization. For electromagnetic simulations, a finite-difference time-domain (FDTD) solver was first developed to compute frequency-dependent S-parameters and field distributions. The mesh was adaptively refined until the variation in scattering parameters fell below 0.1%. On this basis, a transmission-line-theory-based solution set was integrated to determine the optimal microwave absorption performance: the complex permittivity and permeability extracted from the FDTD solver were used to calculate the input impedance and reflection loss under varying thicknesses, and the Pareto front of optimal absorption-bandwidth trade-offs was obtained by solving for thickness conditions that satisfy impedance matching while maximizing the bandwidth below -10 dB. For multiscale simulations, a concurrent coupling scheme was constructed in which the macroscopic electromagnetic responses from the full-wave solver were passed as boundary conditions to microscopic transport models. At the mesoscopic scale, the motion trajectories of bound-state charges in the interfacial region were calculated from the complex permittivity and the frequency-dependent dielectric response. By solving a Langevin-type equation of motion that incorporates restoring forces and polarization relaxation, time-resolved trajectories under alternating fields were obtained to reveal the contribution of interfacial polarization to dielectric loss.

The following is a part of the code:

**References**

[1] Yuan, H. et al. Transparent Organogel Based on Photopolymerizable Magnetic Cationic Monomer for Electromagnetic Wave Absorbing. *J. Ind. Eng. Chem.* 109, 538-546 (2022).

[2] Zhang, H. et al. Room Temperature Fabrication of an RGO-Fe_3_O_4_ Composite Hydrogel and Its Excellent Wave Absorption Properties. *RSC Adv.* 4, 14441-14446 (2014).

[3] Jiang, M. et al. Highly Flexible Hydrogels with Readily Adjustable Electromagnetic Parameter for Efficient Electromagnetic Wave Absorption. *ACS Appl. Nano Mater.* 7, 16488-16497 (2024).

[4] Tang, Y. et al. Multi-functional Electromagnetic Wave Absorption Hydrogel with Adjustable Absorption Frequency. *Carbon* 238, 120238 (2025).

[5] Zhang F. et al. Polarization-driven Multifunctional Organohydrogels with Strain Sensitivity toward Electromagnetic Wave Absorption. *Nano Res.* 17, 5688-5697 (2024).

[6] Lan, D. et al. Environmentally Adaptable Hydrogels with Highly Entangled Interpenetrating Networks for Efficient Electromagnetic Wave Absorption. *Carbon* 253, 121416 (2026).

[7] Fan, X. et al. Flexible Graphene Oxide Hydrogel with Enhanced Mechanical Properties for Electromagnetic Wave Absorption and Thermal Insulation. *ACS Appl. Nano Mater.* 8, 14113-14120 (2025).

[8] Gao, Y. et al. Hydrogel Infusion Additive Manufacturing of Customized Hierarchical Porous Cobalt/Carbon Composites for Electromagnetic Wave Absorption. *J. Mater. Chem. C* 14, 10175-10191 (2026).

[9] Lv, Y. et al. Ionically conductive NiFe_2_O_4_/CNTs Organohydrogel Composite for Boosting Efficient Electromagnetic Wave Absorption. *J. Mater. Sci. Technol.* 249, 253-263 (2026).

[10] Zhang, C. et al. Ni_x_Co_y_-C/Polyacrylamide Hydrogels Derived from Mixed Metal MOF-74 for Synergistically Enhanced Electromagnetic Wave Absorption and Thermal Conduction Performances. *Chem. Eng. J.* 505, 159506 (2025).

[11] Long, Y. et al. Enhanced Electromagnetic Wave Absorption Performance of Hematite@carbon Nanotubes/Polyacrylamide Hydrogel Composites with Good Flexibility and Biocompatibility. *Adv. Compos. Hybrid Mater.* 6, 173 (2023).

[12] Tao, Y. et al. GO-HNT Framework-based Hydrogels with Efficient Water Evaporation-driven Cooling and Superior Electromagnetic Wave Absorption. *Mater. Horiz.* 12, 7000-7011 (2025).

[13] Cai, J. et al. Magnetic Field Vertically Aligned Co-MOF-74 Derivatives/polyacrylamide Hydrogels with Bifunction of Excellent Electromagnetic Wave Absorption and Efficient Thermal Conduction Performances. *Compos. Part A-appl. S.* 176, 107832 (2024).
